# Inequalities in mammograms and cervical biopsies provision in the public and private sectors of the Brazilian healthcare system and the availability of gynecology and obstetrics specialists

**DOI:** 10.1016/j.clinsp.2026.100876

**Published:** 2026-03-04

**Authors:** Ivan Wilson Hossni Dias, Edmund Chada Baracat, Alicia Matijasevich, César Eduardo Fernandes, Cristiane de Jesus Almeida, Paola Soledad Mosquera, Mário César Scheffer

**Affiliations:** a Faculdade de Medicina da Universidade de São Paulo (FMUSP), São Paulo, SP, Brazil; b Associação Médica Brasileira (AMB), São Paulo, SP, Brazil

**Keywords:** Unified health system, Health insurance plans, Breast cancer, Cervical cancer, Access to health services, Physician distribution

## Abstract

**Background:**

This study aimed to evaluate the provision of mammograms and cervical biopsies in Brazil’s public and private healthcare sectors and the availability of gynecologists and obstetricians*.*

**Methods:**

This ecological study included specialists in gynecology and obstetrics, as well as data on mammograms and cervical biopsy procedures performed in 2024 through the Brazilian Unified Health System (SUS) and private health insurance plans. For each state, ratios of specialist availability and the rate of procedures per 100,000 women were calculated. Sensitivity analysis was performed to calculate the ratio of procedures in women of different age groups, and Spearman’s correlation coefficient was used to assess the association between specialist availability and the rate of procedures performed.

**Results:**

In 2024, health insurance plans performed 15,510.88 mammograms per 100,000 women, almost triple the SUS rate (5,733.93). The private sector performed 426.28 cervical biopsies per 100,000 women, while the public sector conducted only 85.24. The total provision of mammograms varied from 12,691.29 per 100,000 women in São Paulo to 2,702.91 in Pará. For cervical biopsies, the provision ranged from 348.94 per 100,000 women in São Paulo to 21.18 in Roraima. Correlation analysis showed a positive association between specialist availability and procedure volume (mammograms: *r* = 0.74; biopsies: *r* = 0.39; p < 0.001), which remained consistent after sensitivity analysis.

**Conclusion:**

Separate indicators for procedure provision and specialist availability effectively identified regional disparities. These findings can guide policymakers in reducing inequalities in access to early breast and cervical cancer diagnosis.

## Highlights

• Gynecologist and obstetrician availability in Brazil shows significant inequalities.
• Mammography and cervical biopsy service provision shows significant inequalities.
• These inequalities exist across regions and between the public and private sectors.
• The inequalities create barriers to breast and cervical cancer diagnosis.
• The inequalities lead to increased health inequities in North and Northeast Brazil.

## Introduction

The epidemiological patterns of cancers among women vary across regions and change over time.^1^ Among the most common cancers in women, breast cancer has the highest incidence in high-income countries,^2^ whereas cervical cancer ‒ the fourth most common cancer among women globally ‒ is more prevalent in low-income countries.^3^ Both cancers are major contributors to premature mortality and years of healthy life lost,^4^ significantly adding to the global burden of disease in women.^5^

In Brazil, excluding non-melanoma skin cancers, breast cancer is the most frequent malignancy among women, with an incidence rate of 41.89 cases per 100,000 in 2022.^6^ Cervical cancer ranks third in incidence among women, with 15.38 cases per 100,000.^7^

The distribution of these cancers across the national territory is uneven. While breast cancer shows higher incidence rates in the South and Southeast regions, cervical cancer remains more prevalent in the North and Northeast, where mortality rates are still high.^6^^,^^7^ These disparities reflect not only socioeconomic differences and variations in exposure to risk factors but also inequities in access to healthcare services, screening, and early diagnosis.^8^

Multiple factors contribute to cancer-related morbidity and mortality among women. On the one hand, individual behavioral, genetic, and environmental factors influence disease incidence.^9^ On the other hand, structural issues related to healthcare systems, such as service organization, access to specialized medical care, coverage, and quality of care, also play a crucial role.^10^, ^11^, ^12^ Poorly functioning services can delay early detection, leading to late diagnoses and, consequently, worse outcomes and higher mortality rates.^2^

This situation can be improved by reducing inequalities in women’s access to healthcare services, particularly specialized care provided by gynecologists and obstetricians. These specialists are essential for primary prevention ‒ such as counseling on risk factors and recommending Human Papillomavirus (HPV) vaccination ‒ and secondary prevention through screening, diagnostic procedures, and therapeutic interventions for breast and cervical conditions,^13^^,^^14^ thereby facilitating early diagnosis and timely treatment.^8^^,^^15^

This study aims to analyze the availability and distribution of gynecologists and obstetricians in Brazil and the provision of mammograms and cervical biopsy procedures across both the public and private healthcare sectors.

## Material and methods

This is an ecological study that utilized Brazilian states as the unit of analysis. Data on gynecologists and obstetricians were obtained by cross-referencing and analyzing databases from the National Medical Residency Commission (CNRM/MEC) and the Brazilian Medical Association (AMB).^16^ The parameters considered included gender, age, place of residence, and the source of specialist certification (either through completion of a medical residency program accredited by the CNRM or a specialist title granted by the Brazilian Federation of Gynecology and Obstetrics Associations (Febrasgo)/AMB). The Strengthening the Reporting of Observational Studies in Epidemiology (STROBE) checklist was used to report the findings from the study.

Data on the provision of cervical biopsy and mammography procedures in Brazil in 2024 were obtained from two official sources: the Outpatient Information System (SIA) of the Department of Informatics of the Unified Health System (DATASUS)^17^ and the Standard Panel for Supplementary Health Information Exchange (TISS) of the National Supplementary Health Agency (ANS).^18^ The selected procedures included cervical biopsy (SUS code: 0201010666; ANS code: 31303021) and mammography (SUS codes: 0204030030, 0204030048, and 0204030188; ANS codes: 40808033, 40808041, and 40808173). The data supporting the findings of this study are available from the corresponding author upon reasonable request.

To assess the relationship between the number of physicians or procedures and the study population, rates per 100,000 were calculated using population data from the 2022 update of the demographic census conducted by the Brazilian Institute of Geography and Statistics (IBGE).^19^

The total population of women was used as the denominator due to the lack of consensus in the literature regarding the age ranges for which the procedures are indicated.^20^, ^21^, ^22^ The authors performed a sensitivity analysis using the female population aged 20–69 years for cervical biopsies, and 50–69 years for mammograms, in consonance with other studies that review screening programs^20^, ^23^ and provision of health services.^24^

Information about the number of women who are beneficiaries of private health insurance plans was obtained from the ANS database.^25^ The population utilizing the *Brazilian Unified Health System* (SUS) was estimated by subtracting the number of women covered by private health insurance from the total population of women.

Spearman’s correlation coefficient was determined to assess the association between the rate of gynecologists and obstetricians and the rate of cervical biopsy and mammography procedures, both per 100,000 women. The significance level was set at 5%.

For each state, this study considered a combination of five indicators of specialist availability or procedure provision: a) The rate of gynecologists and obstetricians per 100,000 women, compared to the national rate; b) The rate of cervical biopsies per 100,000 women, compared to the national rate; c) The rate of mammograms per 100,000 women, compared to the national rate; d) The percentage difference between the rates of mammograms covered by private health insurance plans and the SUS, relative to the national difference; and e) The percentage difference between the rates of cervical biopsies covered by private health insurance plans and the SUS, relative to the national difference.

This study is part of the “Medical Demography in Brazil” project and received approval from the Research Ethics Committee of the University of São Paulo Medical School (CAAE n° 71626323.8.0000.0068).

## Results

In 2024, Brazil had 35,528 physicians specialized in gynecology and obstetrics, representing nearly 10% of all medical specialists in the country (353,287 physicians). The majority were women (63.4%), with a mean age of 51.3-years (SD = 14.1) and a median age of 50.2-years (Table 1).

**Table 1:** Gynecology and obstetrics (OB-GYN) specialists, according to selected parameters in Brazil in 2024.

Parameter	N	%
**Gender** (n = 35.528)		
Women	22,529	63.4
Men	12,999	36.6
**Age (years)** (n = 35.528)		
≤ 35	5,364	15.1
35–50	12,271	34.5
≥ 50	17,893	50.4
**Physician location by municipal population size (inhabitants)^a^** (n = 38.511)		
> 500.000	23,543	61.1
100,000–500.000	9,361	24.3
50.000–100,000	2,474	6.4
20.000–50.000	2,271	5.9
10.000–20.000	605	1.6
< 10.000	257	0.7
**Type of medical school attended^b^** (n = 33.415)		
Private	16,466	49.3
Public	16,949	50.7
**Source of specialist certification** (n = 35.528)		
Medical residency	25,698	72.3
Brazilian Medical Association	9,830	27.7

Source: Scheffer, M. (coord.). Medical Demography in Brazil; Brazilian Institute of Geography and Statistics; National Medical Residency Commission; Brazilian Medical Association.1

Note: a This analysis used the number of specialist registrations (38,511). A total of 35,528 individuals were certified in gynecology and obstetrics, of whom 2,983 were registered in more than one state. b Data loss <10%.

Regarding training, 72.3% had completed a Medical Residency (MR) program, while 27.7% had not undertaken residency but had obtained a specialist title from Febrasgo/AMB. Regarding undergraduate medical education, 50.7% had graduated from public medical schools and 49.3% from private institutions.

Geographically, 61.1% of specialists were concentrated in 48 municipalities with over 500,000 inhabitants, whereas the 4,895 municipalities with fewer than 50,000 residents accounted for approximately 15% of all gynecologists and obstetricians (Table 1).

### Availability of gynecologists and obstetricians

**Figure. 1:**
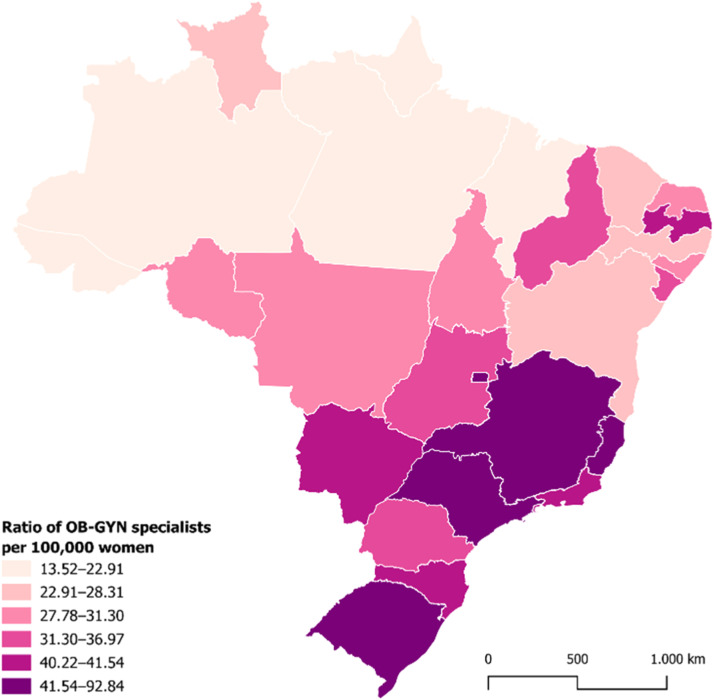
Ratio of Gynecology and Obstetrics (OB-GYN) specialists per 100,000 women, by state in Brazil in 2024. Source: Scheffer, M. (coord.). Medical Demography in Brazil; Brazilian Institute of Geography and Statistics; National Medical Residency Commission; Brazilian Medical Association.1.

The distribution of gynecologists and obstetricians across Brazil was found to be uneven. In 17 states, the rate of specialists fell below the national average of 36.84 per 100,000 women. The North (20.11 per 100,000) and Northeast (25.53 per 100,000) regions presented substantially lower densities compared to the Central-West (45.00), South (42.60), and Southeast (43.85) regions (Fig. 1 and Table 2).

**Table 2:** Gynecology and obstetrics (OB-GYN) specialists and ratio of specialists per 100,000 women, by major regions and states in Brazil in 2024.

Major region/State	OB-GYN specialists^a^	Population of women	Ratio per 100,000 women^b^
**North Region**	**1,748**	**8,691,765**	**20.11**
Acre	94	414,686	22.67
Amapá	77	369,243	20.85
Amazonas	397	1,975,803	20.09
Pará	613	4,068,318	15.07
Rondônia	250	793,209	31.52
Roraima	89	316,315	28.14
Tocantins	228	754,191	30.23
**Northeast Region**	**7,211**	**28,240,713**	**25.53**
Alagoas	479	1,630,264	29.38
Bahia	1,709	7,305,940	23.39
Ceará	1,154	4,537,030	25.44
Maranhão	466	3,447,276	13.52
Paraíba	843	2,055,832	41.01
Pernambuco	1,170	4,737,611	24.70
Piauí	531	1,670,597	31.79
Rio Grande do Norte	491	1,703,967	28.82
Sergipe	368	1,152,196	31.94
**Central-West Region**	**3,727**	**8,282,055**	**45.00**
Federal District	1,369	1,474,595	92.84
Goiás	1,265	3,589,554	35.24
Mato Grosso	516	1,817,408	28.39
Mato Grosso do Sul	577	1,400,498	41.20
**Southeast Region**	**19,285**	**43,980,290**	**43.85**
Espírito Santo	912	1,963,649	46.44
Minas Gerais	4,382	10,524,280	41.64
Rio de Janeiro	3,484	8,477,499	41.10
São Paulo	10,507	23,014,862	45.65
**South Region**	**6,540**	**15,353,502**	**42.60**
Paraná	2,336	5,867,030	39.82
Rio Grande do Sul	2,609	5,627,214	46.36
Santa Catarina	1,595	3,859,258	41.33
**Brazil**	**38,511**	**104,548,325**	**36.84**

Source: Scheffer, M. (coord.). Medical Demography in Brazil; Brazilian Institute of Geography and Statistics; National Medical Residency Commission; Brazilian Medical Association.1

Note: a This analysis used the number of OB-GYN specialist registrations (38,511). b Ratio obtained by dividing the total number of OB-GYN specialists in the state by the population of women estimated based on the 2022 IBGE Census, per 100,000 women.

The Federal District had the highest concentration of gynecologists and obstetricians (92.84 per 100,000 women), followed by Espírito Santo (46.44) and São Paulo (45.65). At the other end of the spectrum, the lowest rates of gynecology and obstetrics specialists were observed in Amazonas (20.09), Pará (15.07), and Maranhão (13.52).

### Provision of mammograms

Marked inequalities were observed in the distribution of mammograms, both across regions and states and between public (SUS) and private (health insurance) sectors (Table 3).

**Table 3:** Mammogram procedures in Brazil, covered by the Unified Health System (SUS) and private health insurance plans, by major regions and states in Brazil in 2024.

	Ratio per 100,000 women (Private health insurance)	Ratio per 100,000 women (SUS)	Ratio per 100,000 women (Total)	Δ%^a^
**North Region**	**9,311.27**	**2,060.74**	**2,908.79**	**351.8**
Acre	11,419.11	4,412.57	4,836.19	158.8
Amapá	12,018.88	2,047.04	2,973.92	487.2
Amazonas	6,540.67	2,030.85	2,812.27	222.1
Pará	10,799.34	1,702.41	2,702.91	534.5
Rondônia	10,492.86	2,575.59	3,443.99	307.3
Roraima	8,742.31	4,486.72	4,709.86	94.8
Tocantins	9,993.42	1,110.31	1,862.26	800.3
**Northeast Region**	**14,001.92**	**4,426.21**	**5,755.63**	**216.4**
Alagoas	15,238.34	4,355.76	5,745.20	249.8
Bahia	13,190.84	6,170.52	7,037.97	113.8
Ceará	13,910.48	3,330.11	5,149.03	317.7
Maranhão	9,035.52	1,937.70	2,469.28	366.3
Paraíba	15,552.94	3,623.22	5,073.52	329.3
Pernambuco	14,984.68	3,958.36	5,775.70	278.6
Piauí	14,382.59	5,639.32	6,791.52	155.1
Rio Grande do Norte	15,581.18	5,492.24	7,516.69	183.7
Sergipe	14,279.16	5,530.52	6,888.41	158.2
**Central-West Region**	**10,597.03**	**3,759.27**	**5,613.18**	**181.9**
Federal District	12,317.32	2,544.66	6,042.00	384.0
Goiás	7,939.17	3,991.93	5,121.58	98.9
Mato Grosso	13,594.33	2,617.34	4,654.71	419.4
Mato Grosso do Sul	12,894.69	5,902.00	7,665.49	118.5
**Southeast Region**	**15,971.25**	**7,889.46**	**10,897.53**	**102.5**
Espírito Santo	16,397.21	7,673.49	10,640.95	113.7
Minas Gerais	15,636.53	5,375.76	8,318.97	190.9
Rio de Janeiro	16,014.01	5,550.65	9,288.38	188.5
São Paulo	16,033.55	10,276.33	12,691.29	56.0
**South Region**	**19,499.76**	**6,820.19**	**10,073.17**	**185.9**
Paraná	19,652.36	7,106.12	10,666.10	176.6
Rio Grande do Sul	19,796.15	7,587.46	10,598.85	160.9
Santa Catarina	18,748.88	5,322.02	8,405.27	252.3
**Brazil**	**15,510.88**	5,733.93	**8,304.77**	**170.5**

Source: National Supplementary Health Agency; DATASUS; Brazilian Institute of Geography and Statistics. Note: a Percentage difference between the ratio of mammograms per 100,000 women covered by private health insurance plans and the ratio of mammograms per 100,000 women covered by the SUS, multiplied by 100.

The SUS provided 51% of all mammograms conducted in Brazil, corresponding to a rate of 5,733.93 procedures per 100,000 women who rely exclusively on the public system. Among women covered by private health insurance plans, who represent approximately one-quarter of the Brazilian population, the rate was 15,510.88 mammograms per 100,000 women ‒ approximately three times higher than that observed in the SUS. In absolute terms, private health plans accounted for 49% of the mammograms conducted in 2024.

These disparities were also found to manifest regionally: while the Southeast region conducted 10,897.53 mammograms per 100,000 women, the North performed only 2,908.79. In every state, rates were higher in the private sector than in the SUS. The greatest discrepancies were observed in Tocantins, where private plans were responsible for 800.3% more mammograms than the public system, followed by Pará (534.5%) and Amapá (487.2%).

### Provision of cervical biopsies

As with mammograms, the provision of cervical biopsies revealed inequalities across regions, states, and healthcare sectors. The SUS accounted for 35.9% of all cervical biopsies conducted in Brazil, equivalent to 85.24 procedures per 100,000 women who rely on the public system. Private health insurance plans were responsible for 64.1% of the biopsies. Among women with private coverage, the rate was 426.28 cervical biopsies per 100,000 women, approximately five times higher than that observed in the SUS, when considering the size of the population served (Table 4).

**Table 4:** Cervical biopsy procedures in Brazil, covered by the Unified Health System (SUS) and private health insurance plans, by major regions and states in Brazil in 2024.

	Ratio per 100,000 women (Private health insurance)	Ratio per 100,000 women (SUS)	Ratio per 100,000 women (Total)	Δ%^a^
**North Region**	**90.50**	**59.26**	**62.91**	**52.7**
Acre	223.36	148.87	153.37	50.0
Amapá	777.95	42.70	111.04	1,721.9
Amazonas	4.67	78.06	65.34	-94.0
Pará	74.42	56.51	58.48	31.7
Rondônia	85.06	41.35	46.14	105.7
Roraima	54.26	19.35	21.18	180.4
Tocantins	258.45	22.31	42.30	1,058.4
**Northeast Region**	**371.28**	**80.89**	**121.21**	**359.0**
Alagoas	1,008.91	87.26	204.94	1,056.2
Bahia	156.52	116.61	121.54	34.2
Ceará	280.90	47.70	87.79	488.9
Maranhão	170.42	65.97	73.80	158.3
Paraíba	914.27	85.05	185.86	975.0
Pernambuco	492.93	82.01	149.74	501.1
Piauí	701.78	36.95	124.57	1,799.3
Rio Grande do Norte	137.17	21.07	44.37	551.0
Sergipe	148.18	150.51	150.15	-1.5
**Central-West Region**	**247.16**	**68.27**	**116.77**	**262.0**
Federal District	307.55	100.75	174.76	205.3
Goiás	217.17	26.81	81.29	710.0
Mato Grosso	238.06	105.47	130.08	125.7
Mato Grosso do Sul	252.84	87.75	129.38	188.1
**Southeast Region**	**472.92**	**92.58**	**234.14**	**410.8**
Espírito Santo	145.37	111.83	123.24	30.0
Minas Gerais	164.77	84.01	107.17	96.1
Rio de Janeiro	189.54	59.24	105.79	220.0
São Paulo	680.83	109.13	348.94	523.9
**South Region**	**476.01**	**103.16**	**198.81**	**361.4**
Paraná	247.30	161.08	185.55	53.5
Rio Grande do Sul	926.51	60.95	274.45	1,420.1
Santa Catarina	200.06	81.47	108.70	145.6
**Brazil**	**426.28**	**85.24**	**174.91**	**400.1**

Source: National Supplementary Health Agency; DATASUS; Brazilian Institute of Geography and Statistics. Note: a Percentage difference between the ratio of cervical biopsies per 100,000 women covered by private health insurance plans and the ratio of cervical biopsies per 100,000 women covered by the SUS, multiplied by 100.

Comparisons across major regions showed that the Southeast performed 234.14 procedures per 100,000 women, while the North region conducted only 62.91. When comparing the public and private sectors, more procedures were covered by private health plans than the SUS in all states, excluding Amazonas and Sergipe. The most striking discrepancies occurred in Piauí, where private plans were responsible for 1,799.3% more cervical biopsies than the public system, followed by Amapá (1,721.9%) and Tocantins (1,058.4%).

### Sensitivity analysis

Among women aged 50 to 69, the private sector performed more procedures nationally (36,485 per 100,000 women) than SUS (16,770), with states like São Paulo and Bahia showing strong public sector performance (Supplementary Table 1). When considering women of all ages, private health insurance plans dominated, with a national rate of 15,510 per 100,000 women compared to just 5,733 in the SUS, resulting in a percentage difference of 170.5% (Table 3).

For cervical biopsies, the pattern remained consistent with the rate of procedures in the private sector (625.73 per 100,000 women), significantly higher than in the public system (122.86), resulting in a percentage difference of 409.3%. This trend was consistent across most regions, with particularly contrasting in the Northeast, Southeast, and South. States such as Piauí, Alagoas, and Rio Grande do Sul exhibited extreme reliance on private coverage. Conversely, Amazonas and Sergipe were the only states where SUS performed more biopsies than the private sector. Alagoas stood out with the highest absolute rate of private procedures (1,482.48 procedures per 100,000 women aged 50‒69 yr-old), while Amapá showed the highest difference between public and private sectors (1,566.8%) (Supplementary Table 2).

### Correlation analysis

The study also analyzed the correlation between the distribution of gynecologists and obstetricians and the rates of cervical biopsies and mammograms.

The Spearman’s correlation analysis showed a positive association between the rate of mammograms (*r* = 0.74; p < 0.001) and biopsies (*r* = 0.44; p < 0.001) and the availability of specialists. In the sensitivity analysis, the pattern remained consistent, with a positive association between the rate of procedures (mammograms: *r* = 0.66; p < 0.001; biopsies: 0.46; p < 0.001) and the rate of specialists (data not presented in the Table).

### Indicators of physician availability and procedure provision

The availability of professionals and the provision of mammograms and cervical biopsies in Brazil were characterized using five indicators: the number of gynecologists and obstetricians per 100,000 women; the total number of mammograms and cervical biopsies per 100,000 women; and the percentage difference in the rates of these procedures covered by private health insurance plans compared to the SUS.

A state’s performance was classified as positive (+) when it was above the national average for a given indicator and negative (–) when it was below that average (Table 5). For the indicators comparing private plans and the SUS, values above the national difference were classified as positive (+) and those below as negative (–).

**Table 5:** Indicators of the availability and provision of mammogram and cervical biopsy services by major regions and states in Brazil in 2024.

Region/State	OB-GYN specialists per 100,000 women^(a)^	Mammograms per 100,000 women^(^^b^^)^	Cervical biopsies per 100,000 women^(^^c^^)^	Difference between the SUS and private health insurance for mammograms^(d)^	Difference between the SUS and private health insurance for cervical biopsies^(e)^
**North Region**	**-**	**-**	**-**	**+**	**-**
Acre	-	-	-	**-**	-
Amapá	-	-	-	**+**	+
Amazonas	-	-	-	**+**	-
Pará	-	-	-	**+**	-
Rondônia	-	-	-	**+**	-
Roraima	-	-	-	**-**	-
Tocantins	-	-	-	**+**	+
**Northeast Region**	**-**	**-**	**-**	**+**	-
Alagoas	-	-	**+**	**+**	+
Bahia	-	-	-	**-**	-
Ceará	-	-	-	**+**	+
Maranhão	-	-	-	**+**	-
Paraíba	+	-	+	**+**	+
Pernambuco	-	-	-	**+**	+
Piauí	-	-	-	**-**	+
Rio Grande do Norte	-	-	-	**+**	+
Sergipe	-	-	**-**	**-**	-
**Central-West Region**	**+**	**-**	**-**	**+**	**-**
Federal District	+	-	-	**+**	-
Goiás	-	-	-	**-**	+
Mato Grosso	-	-	-	**+**	-
Mato Grosso do Sul	+	-	-	**-**	-
**Southeast Region**	**+**	**+**	**+**	**-**	**+**
Espírito Santo	+	**+**	-	**-**	-
Minas Gerais	+	**+**	-	**+**	-
Rio de Janeiro	+	**+**	-	**+**	-
São Paulo	+	**+**	**+**	**-**	**+**
**South Region**	**+**	**+**	**+**	**+**	**-**
Paraná	+	**+**	**+**	**+**	-
Rio Grande do Sul	+	**+**	**+**	**-**	+
Santa Catarina	+	**+**	-	+	-

a Positive (+): above 36.84 OB-GYN specialists per 100,000 women. Negative (-): below 36.84 per 100,000 women.

b Positive (+): above 8,304.77 mammograms per 100,000 women. Negative (-): below 8,304.77 per 100,000 women.

c Positive (+): above 174.91 cervical biopsies per 100,000 women. Negative (-): below 174.91 per 100,000 women.

d Positive (+): above 170.5%. Negative (-): below 170.5%.

e Positive (+): above 400.1%. Negative (-): below 400.1%.

Note: OB-GYN, Obstetrics and Gynecology.

The North and Northeast regions had the highest number of states with below-average performance in terms of the availability of gynecologists and obstetricians and the total number of procedures per 100,000 women, in addition to percentage differences between the private and public sectors that exceeded the national average. Except for Paraíba, all states in these two regions had a lower-than-average supply of gynecologists and obstetricians compared with the national rate of 36.84 specialists per 100,000 women. In the Central-West region, only Goiás (35.24) and Mato Grosso (28.39) were below this average.

Regarding mammogram volume, states in the Southeast and South exceeded the national average of 8,304.77 procedures per 100,000 women, with notable figures in São Paulo (12,691.29) and Espírito Santo (10,640.95), while Tocantins (1,862.26) and Maranhão (2,468.28) had the lowest mammogram rates in the country. As for cervical biopsies, Paraíba (185.86), Alagoas (204.91), São Paulo (348.94), Paraná (185.55), and Rio Grande do Sul (274.45) surpassed the national average of 174.91 procedures per 100,000 women, with Roraima (21.18) and Tocantins (42.30) showing the lowest volumes.

The percentage differences in mammogram rates between private health plans and the SUS were significantly higher in the North and Northeast regions, surpassing the national average of 170.5%, with the highest disparities in Amapá (487.2%), Pará (534.5%), Tocantins (800.3%), Maranhão (366.3%), and Paraíba (329.3%). Conversely, São Paulo (56%) and Roraima (94.8%) had the lowest differences. When considering women aged 50 to 69, most states in the North and Northeast regions show higher private sector mammography rates compared to SUS. In the Centre-West, Southeast and South regions, the highest disparities were observed in states of Goiás (391.2%), Minas Gerais (145.8%), and Santa Catarina (237.5%).

For cervical biopsies, eleven states exceeded the national average of 400.1% in the percentage difference between private health plans and the SUS, with the highest disparities observed in Amapá (1,721.9%), Tocantins (1,058.4%), Piauí (1,799.3%), and Alagoas (1,056.2%). Only Amazonas and Sergipe presented negative percentage differences, indicating a higher rate of procedures covered by the SUS than by private health plans. The overall pattern remained consistent when considering women aged 20 to 69-years, with the exception of Amazonas (-94.8%) and Sergipe (-2.7%), which showed a positive difference favoring SUS.

Except for Espírito Santo and Santa Catarina, all other states in the South and Southeast regions had two or fewer indicators below the national average. No state exhibited all three availability indicators above the national average while simultaneously showing both percentage-difference indicators (comparing private plans and the SUS) below the national average (Table 5).

## Discussion

This study is original in jointly analyzing the provision of crucial screening procedures for cervical and breast cancer and the availability of gynecologists and obstetricians, considering two dimensions of inequality within the Brazilian healthcare system: territorial discrepancies and disparities between the public and private sectors.

Health inequalities become inequities when they are systematic, avoidable, and unjust.^26^ The present findings illustrate the overlapping nature of structural inequities across public-private services and regional disparities in the provision of diagnostic exams. These results complement previous studies that have examined territorial inequalities in Brazil,^24^ particularly those addressing access to gynecological services.^24^^,^^28^ The unequal distribution of gynecologists and diagnostic services in affluent areas may reflect the persistent healthcare inequities, as it shows that medical services are least available where they are most needed, the so-called “inverse care law”.^29^

Several mechanisms are proposed to explain how the unequal distribution of physicians translates into unequal provision of diagnostic services. First, physicians tend to cluster in areas with greater infrastructure and higher density of hospitals and clinics, which in turn expands the supply of diagnostic exams.^30^ The presence of specialists fosters diagnostic capacity, attracting investments in equipment and creating a reinforcing cycle that strengthens already privileged regions. Second, economic and institutional incentives, including better remuneration and working conditions in wealthier areas, are often linked to the coexistence of a strong private sector, further accentuating regional concentration of the medical workforce.^30^ Higher physician density can also reduce access barriers, such as waiting and referral times, increasing the likelihood that women in better-resourced regions will receive timely diagnostic care.^31^

The finding that private health plans covered substantially more procedures per 100,000 women in 2024 than the public system aligns with previous reports indicating that women with private health coverage have greater access to cancer diagnostic procedures, even after adjusting for socioeconomic status.^32^ This interpretation, however, of procedure rates requires caution. Indicators based on the number of procedures performed reflect health service provision, which does not necessarily correspond to access or met need.

Higher rates of mammograms in the private sector may indicate expanded access, but could also reflect overuse associated with fee-for-service incentives. Similarly, low rates of cervical biopsies may result from either efficient screening programs or from system failures in screening or referral. The extremely low rate observed in some North and Northeast states, like Roraima and Rio Grande do Norte, is more likely to reflect barriers in service provision rather than a lower population need, given the high burden of cervical cancer in the region. Therefore, these ecological correlations should not be interpreted as a simple “more is better” pattern, but rather as markers of complex and context-dependent care pathways.

Persistence of health inequalities has been studied in Brazil in the last decade.^24^^,^^27^^,^^28^^,^^31^ Without deliberate redistribution of resources, workforce, and decision-making power, universal health systems can exacerbate disparities ‒ particularly when public and private sectors coexist without integration.^33^^,^^34^ Firstly, because private financing, user fees, or tiered benefits allow higher-income groups to secure faster or superior care. Second, specialized and high-quality services tend to concentrate in areas with better infrastructure and socioeconomic conditions, limiting effective access for disadvantaged groups.^29^^,^^30^ Finally, because new benefits or coverage expansions are initially accessed by wealthier or better-informed groups, temporarily widening disparities before poorer populations catch up – the so-called “inverse equity effect”.^35^

The findings presented here, which highlight structural asymmetries in cancer care driven by the availability of human resources and diagnostic procedures, contribute to reframing^33^ the measurement of inequality, which is typically focused on social determinants and health outcomes such as morbidity and mortality. Fragmented health systems with a high proportion of private spending are known to report lower screening coverage and poorer indicators of cancer diagnosis and mortality, particularly for breast and cervical cancer.^36^^,^^37^

The correlation analysis showed a positive association between the rate of gynecologists and obstetricians, as well as the performance of mammograms and cervical biopsies, consistent with outcomes from France and the Balkan region.^15^^,^^38^ However, after sensitivity analysis for different population age groups, the correlation decreased, particularly for mammograms, indicating the influence of factors related to service organization, screening policies, and patient demand.

As observed for the overall physician workforce and other specialties,^16^ gynecologists and obstetricians are mainly concentrated in large urban centers and in the South and Southeast regions. This workforce largely comprises women (63.4%), making it the seventh specialty with the highest proportion of women after dermatology (80.6%) and pediatrics (76.8%).^16^

It must be noted that the presence of gynecologists alone does not guarantee the provision of procedures, which also depends on equipment availability, diagnostic infrastructure, the size of the private healthcare market, and referral flows between services and care levels. This may help explain cases in which states with similar physician-to-population ratios, such as São Paulo (45.65 physicians per 100,000 women) and Espírito Santo (46.44 physicians per 100,000 women), presented significant differences in examination rates. São Paulo recorded 12,691.29 mammograms and 348.94 cervical biopsies per 100,000 women, whereas Espírito Santo reported 10,640.95 mammograms and only 123.24 cervical biopsies per 100,000 women.

The correlation analyses for biopsies indicate that the performance of this procedure depends on earlier stages of care and not solely on the availability of specialists. For a biopsy to be conducted, a woman must have previously undergone a screening test that identified an abnormality, followed by a suitable medical referral and the availability of pathologists for diagnostic confirmation. Thus, the provision of biopsies is conditioned by a sequence of clinical and organizational factors, which may explain the variability observed across Brazilian states. After conducting a sensitivity analysis, the correlation remained virtually unchanged with the inclusion of age groups from 20 to 69-year range, which encompasses the majority of screening and diagnostic activities. This suggests that the influence of very young or elderly women on the results is limited, possibly due to their lower frequency of examinations or reduced inclusion in standard clinical protocols.

For mammography, which is part of more established screening programs within the SUS, sensitivity analysis showed that while SUS prioritizes screening within the recommended age group, access to mammography outside this range can be largely dependent on private coverage. States such as Tocantins and Amapá showed high levels of inequality in both age groups, whereas São Paulo displayed a smaller relative gap in the recommended age group.

The use of multiple supply indicators, combining physician availability and diagnostic procedures in both absolute terms and differences between public and private networks, proved effective in identifying areas and potential levels of insufficient care across regions and states.

States in the North and Northeast, characterized simultaneously by a lower concentration of gynecologists and obstetricians, lower volumes of mammograms and cervical biopsies, and greater discrepancies between the public and private sectors, are also those where other studies have reported disparities in the accessibility to cancer treatment,^31^ a higher rate of cervical cancer occurence;^7^ higher breast cancer mortality;^6^ and lower cytopathologic testing coverage,^39^ HPV vaccination,^40^ and mammograms.^28^ Therefore, these states should be prioritized in the adoption of emergency measures to expand access to specialists and strengthen cancer screening. In the South and Southeast regions, although indicators are relatively better, screening rates remain below international recommendations.^6^

The overlap of geographic and public-private inequalities in the availability of physicians and diagnostic tests, in a context where waiting times for mammograms and cervical biopsies remain a major barrier to access within the SUS, deserves particular attention, as it produces significant inequities. Shifting part of the current supply and service delivery ‒ now concentrated in the private sector ‒ into the SUS will require the involvement of federal, state, and municipal authorities, along with increased public funding, as well as regulation of procedure costs and professional practices.

The present study has certain drawbacks. Among 35,528 physicians, 2,983 are registered in more than one Regional Medical Council (CRM). For this group, the study considered the same physician in each state in which they were registered. Second, all gynecologists and obstetricians were assumed to be capable of performing the procedures analyzed, although some may primarily engage in other activities or practice in other specialties for which they are also certified. The ecological design precludes inferences at the individual level, and the observed associations may not reflect causal relationships. By its aggregated nature, the study does not allow us to infer whether the women who consult gynecologists are the same ones who undergo the procedures. Another important limitation relates to the potential underreporting of data in both information systems used. In the public sector, DATASUS depends on accurate reporting by health facilities, which may vary across regions. In the private sector, ANS data are often incomplete, particularly regarding procedure counts and professional registration. Such underreporting could result in underestimation of the availability of specialists or diagnostic procedures in certain states, thereby affecting the magnitude, and possibly the direction, of the observed associations. The findings should be interpreted with caution, as variations in data completeness between the public (DATASUS) and private (ANS) systems may influence the apparent regional differences. Improved data integration and reporting quality are essential to ensure more accurate monitoring of healthcare inequalities in Brazil. Finally, the national averages used as benchmarks to assess physician and diagnostic test availability across states should be interpreted with caution, as no internationally established reference values exist for these indicators.

## Conclusions

This study identified significant inequalities in the availability of gynecologists and obstetricians, as well as in the provision of mammography and cervical biopsy services in Brazil, both across regions and between the public and private sectors. These disparities create barriers to early diagnosis and perpetuate health inequities, particularly in the North and Northeast regions. The findings underscore the need for urgent strategies to expand access to specialists and diagnostic services, alongside increased public funding and stronger regulation of the private sector, to ensure that early cancer diagnosis is accessible to all women as a fundamental right.

## Funding

Funding was provided by the São Paulo Research Foundation [FAPESP, grant number 2023/10124-0]. Additional support was provided through Agreement Letter No. SCON2023-00159, established among the Fundação Faculdade de Medicina (FFM), the Pan American Health Organization (PAHO/WHO Brazil), and the Brazilian Ministry of Health (MS), as well as through a Technical Cooperation Agreement between the University of São Paulo (USP) and the Brazilian Medical Association (AMB) (USP Agreement n° 1014318). Ivan Wilson Hossni Dias [grant 2024/22867-0] and Paola Mosquera [grant number 2024/17428-8] received a postdoctoral fellowship from FAPESP as part of the ‘Programa de Pesquisa em Políticas Públicas’.

## Data availability statement

The datasets generated and/or analyzed during the current study are available from the corresponding author upon reasonable request.

## Declaration of competing interest

The authors declare no conflicts of interest.

## Appendix Supplementary materials

Image, application 1

## Footnotes

Supplementary material associated with this article can be found, in the online version, at doi:10.1016/j.clinsp.2026.100876.
