# Supplementary material for: Inequalities in mammograms and cervical biopsies provision in the public and private sectors of the Brazilian healthcare system and the availability of gynecology and obstetrics specialists
Source: Clinics (Sao Paulo). 2026 Mar 4;81:100876. doi: 10.1016/j.clinsp.2026.100876 (PMC12969789; doi:10.1016/j.clinsp.2026.100876)
Supplement: Supplementary file 1 [file mmc1.docx]

CLINICS-D-25-01529_Supplementary Material

**SENSISTIVITY ANALYSIS**

**Supplementary Table 1** Mammogram procedures in Brazil, covered by the Unified Health System (SUS) and private health insurance plans, by major regions and states in Brazil in 2024.

|  | **Ratio per 100,000 women 50 – 69 yr (Private health insurance)** | **Ratio per 100,000 women 50 – 69 yr (SUS)** | **Ratio per 100,000 women 50 – 69 yr (Total)** | **Δ%^a^** |
| --- | --- | --- | --- | --- |
| **North Region** | **26,435.13** | **7,953.18** | **10,192.82** | **232.4%** |
| Acre | 28,428.83 | 15,240.14 | 16,352.83 | 86.5% |
| Amapá | 30,856.88 | 8,363.36 | 11,184.18 | 269.0% |
| Amazonas | 17,029.23 | 9,760.72 | 11,194.48 | 74.5% |
| Pará | 32,075.99 | 6,861.21 | 9,592.08 | 367.5% |
| Rondônia | 30,875.28 | 7,691.67 | 10,113.75 | 301.4% |
| Roraima | 24,050.19 | 17,064.39 | 17,550.71 | 40.9% |
| Tocantins | 29,380.83 | 3,743.07 | 5,573.82 | 684.9% |
| **Northeast Region** | **36,018.37** | **14,457.01** | **17,119.10** | **149.1%** |
| Alagoas | 36,823.73 | 14,547.01 | 17,301.67 | 153.1% |
| Bahia | 30,544.80 | 20,017.14 | 21,225.37 | 52.6% |
| Ceará | 39,602.68 | 10,577.57 | 14,583.22 | 274.4% |
| Maranhão | 26,905.92 | 6,878.21 | 8,160.87 | 291.2% |
| Paraíba | 40,702.85 | 10,870.28 | 14,081.01 | 274.4% |
| Pernambuco | 37,706.97 | 13,689.17 | 17,184.90 | 175.5% |
| Piauí | 44,318.78 | 17,585.47 | 20,188.77 | 152.0% |
| Rio Grande do Norte | 37,509.70 | 16,088.51 | 20,166.55 | 133.1% |
| Sergipe | 35,040.79 | 16,438.20 | 19,068.14 | 113.2% |
| **Central-West Region** | **24,723.51** | **11,601.16** | **15,072.39** | **113.1%** |
| Federal District | 31,946.35 | 16,858.96 | 20,505.36 | 89.5% |
| Goiás | 42,042.25 | 8,558.72 | 13,662.17 | 391.2% |
| Mato Grosso | 17,153.88 | 11,983.96 | 13,454.92 | 43.1% |
| Mato Grosso do Sul | 26,574.05 | 9,047.79 | 15,385.68 | 193.7% |
| **Southeast Region** | **36,579.77** | **20,460.14** | **25,557.15** | **78.8%** |
| Espírito Santo | 37,804.78 | 20,508.44 | 25,306.04 | 84.3% |
| Minas Gerais | 37,269.72 | 15,159.97 | 20,385.23 | 145.8% |
| Rio de Janeiro | 33,448.29 | 15,525.45 | 21,456.12 | 115.4% |
| São Paulo | 37,431.42 | 25,257.73 | 29,519.86 | 48.2% |
| **South Region** | **45,666.78** | **17,521.98** | **23,283.65** | **160.6%** |
| Paraná | 47,081.04 | 18,786.54 | 25,396.05 | 150.6% |
| Rio Grande do Sul | 42,293.69 | 18,263.30 | 22,959.35 | 131.6% |
| Santa Catarina | 48,879.68 | 14,483.43 | 20,481.32 | 237.5% |
| **Brazil** | **36,485.55** | **16,770.67** | **21,401.37** | **117.6%** |

**Source:** National Supplementary Health Agency; DATASUS; Brazilian Institute of Geography and Statistics. **Note:** ^a^Percentage difference between the ratio of mammograms per 100,000 women 50–69-year-old covered by private health insurance plans and the ratio of mammograms per 100,000 women 50–69-year-old covered by the SUS, multiplied by 100.

**Supplementary Table 2** Cervical biopsy procedures in Brazil, covered by the Unified Health System (SUS) and private health insurance plans, by major regions and states in Brazil in 2024.

|  | **Ratio per 100,000 women 20 – 69 yr (Private health insurance)** | **Ratio per 100,000 women 20 – 69 (SUS)** | **Ratio per 100,000 women 20 – 69 (Total)** | **Δ%^a^** |
| --- | --- | --- | --- | --- |
| **North Region** | **130.37** | **92.27** | **97.07** | **41.3%** |
| Acre | 331.21 | 242.41 | 248.28 | 36.6% |
| Amapá | 1,139.48 | 68.36 | 175.69 | 1,566.8% |
| Amazonas | 6.77 | 130.89 | 106.24 | -94.8% |
| Pará | 109.76 | 85.88 | 88.61 | 27.8% |
| Rondônia | 122.10 | 56.05 | 63.50 | 117.8% |
| Roraima | 80.54 | 32.77 | 35.60 | 145.8% |
| Tocantins | 358.85 | 33.58 | 63.50 | 968.5% |
| **Northeast Region** | **532.01** | **119.67** | **179.47** | **344.6%** |
| Alagoas | 1,482.48 | 131.03 | 306.04 | 1,031.4% |
| Bahia | 213.05 | 170.93 | 176.54 | 24.6% |
| Ceará | 398.99 | 69.57 | 128.93 | 473.5% |
| Maranhão | 252.00 | 101.78 | 113.72 | 147.6% |
| Paraíba | 1,355.08 | 127.26 | 278.12 | 964.8% |
| Pernambuco | 723.65 | 120.23 | 221.77 | 501.9% |
| Piauí | 1,068.40 | 53.80 | 187.10 | 1,886.0% |
| Rio Grande do Norte | 193.19 | 30.87 | 64.59 | 525.9% |
| Sergipe | 214.92 | 220.87 | 219.93 | -2.7% |
| **Central-West Region** | **349.95** | **100.15** | **169.89** | **249.4%** |
| Federal District | 362.90 | 132.83 | 192.30 | 173.2% |
| Goiás | 330.92 | 155.99 | 189.69 | 112.1% |
| Mato Grosso | 311.51 | 38.50 | 117.94 | 709.0% |
| Mato Grosso do Sul | 425.16 | 146.80 | 250.11 | 189.6% |
| **Southeast Region** | 668.41 | 132.19 | 333.88 | 405.7% |
| Espírito Santo | 198.28 | 167.15 | 178.51 | 18.6% |
| Minas Gerais | 234.74 | 120.68 | 153.92 | 94.5% |
| Rio de Janeiro | 275.18 | 83.87 | 151.12 | 228.1% |
| São Paulo | 956.33 | 155.29 | 495.25 | 515.9% |
| **South Region** | **664.82** | **148.36** | **285.17** | **348.1%** |
| Paraná | 350.69 | 229.91 | 264.84 | 52.5% |
| Rio Grande do Sul | 1,287.53 | 88.81 | 397.08 | 1,349.7% |
| Santa Catarina | 275.46 | 117.68 | 155.50 | 134.1% |
| **Brazil** | **625.73** | **122.86** | **254.40** | **409.3%** |

**Source:** National Supplementary Health Agency; DATASUS; Brazilian Institute of Geography and Statistics. **Note:** ^a^ Percentage difference between the ratio of cervical biopsies per 100,000 women 20–69-year-old covered by private health insurance plans and the ratio of cervical biopsies per 100,000 women 20–69-year-old covered by the SUS, multiplied by 100.
